# Candidate pathway association and genome‐wide association approaches reveal alternative genetic architectures of carotenoid content in cultivated sunflower (*Helianthus annuus*)

**DOI:** 10.1002/aps3.11558

**Published:** 2023-12-02

**Authors:** Jordan A. Dowell, Chase Mason

**Affiliations:** ^1^ Department of Plant Sciences University of California Davis California 95616 USA; ^2^ Department of Biology University of Central Florida Orlando Florida 32816 USA; ^3^ Present address: Department of Biological Sciences Louisiana State University Baton Rouge Louisiana 70803 USA

**Keywords:** Bayesian sparse, candidate pathway association, carotenoid, GWAS, genomics, linear mixed model

## Abstract

**Premise:**

The explosion of available genomic data poses significant opportunities and challenges for genome‐wide association studies. Current approaches via linear mixed models (LMM) are straightforward but prevent flexible assumptions of an a priori genomic architecture, while Bayesian sparse LMMs (BSLMMs) allow this flexibility. Complex traits, such as specialized metabolites, are subject to various hierarchical effects, including gene regulation, enzyme efficiency, and the availability of reactants.

**Methods:**

To identify alternative genetic architectures, we examined the genetic architecture underlying the carotenoid content of an association mapping panel of *Helianthus annuus* individuals using multiple BSLMM and LMM frameworks.

**Results:**

The LMMs of genome‐wide single‐nucleotide polymorphisms (SNPs) identified a single transcription factor responsible for the observed variations in the carotenoid content; however, a BSLMM of the SNPs with the bottom 1% of effect sizes from the results of the LMM identified multiple biologically relevant quantitative trait loci (QTLs) for carotenoid content external to the known (annotated) carotenoid pathway. A candidate pathway analysis (CPA) suggested a β‐carotene isomerase to be the enzyme with the highest impact on the observed carotenoid content within the carotenoid pathway.

**Discussion:**

While traditional LMM approaches suggested a single unknown transcription factor associated with carotenoid content variation in sunflower petals, BSLMM proposed several QTLs with interpretable biological relevance to this trait. In addition, the CPA allowed for the dissection of the regulatory vs. biosynthetic genetic architectures underlying this metabolic trait.

Genome‐wide association (GWA) research has exploded over the years since the first successful study was published in 2002 (Ozaki et al., 2002), with hundreds of studies published to date. GWA studies (GWAS) frequently use publicly available genomic data, such as single‐nucleotide polymorphism (SNP) data sets. While technological advances have eased access to high‐quality genomic data, phenomics has advanced more slowly (Houle et al., 2010; Furbank and Tester, 2011); however, recent phenomics approaches have revolutionized the GWAS landscape, with studies shifting from analyzing a few phenotypes to hundreds or thousands (York, 2018). With the inclusion of more numerous and complex phenotypes, recent studies have demonstrated complex genomic architectures of traits and their contextual relevance (Kwak et al., 2016; Porter and O'Reilly, 2017).

The primary workhorse of GWAS is the linear mixed model (LMM), popularized by Zhou and Stephens (2012) with the genome‐wide efficient mixed‐model association (GEMMA) method, which is the modern iteration of the Lande and Arnold (1983) multiple regression framework. The LMM incorporates three terms—population structure, kinship, and relevant SNPs—in a GWA framework. Population structure is traditionally estimated in a Bayesian or frequentist framework using the software package STRUCTURE (Pritchard et al., 2000) or a principal component analysis (Zhang et al., 2010; Vilhjálmsson and Nordborg, 2013), while kinship is derived based on various algorithms to reconstruct pedigree information from genomic data (Zhang et al., 2010; Vilhjálmsson and Nordborg, 2013). In practice, these terms are assumed to account for the confounding effects of variance attributed to shared allelic history (Zhang et al., 2010; Vilhjálmsson and Nordborg, 2013); however, population structure and kinship do not fully describe potential genomic background effects (Vilhjálmsson and Nordborg, 2013). To expand the LMM framework, several iterative multi‐locus models have been developed that iteratively incorporate multiple SNPs as individual fixed effects to identify additive epistasis (first‐order effects) (Segura et al., 2012; Liu et al., 2016; Huang et al., 2019).

Behind the Bayesian sparse linear mixed model (BSLMM) popularized in GEMMA (Zhou et al., 2013) is the hypothesis that a SNP can be represented as a unique effect (first‐order effects) plus the effect of a set of loci modifying the SNP's effect (second‐order effects). The integrative epistasis hypothesis of the BSLMM is an alternative to that of the LMM and other popular multi‐locus models (FarmCPU and BLINK), which account for SNPs as singular fixed effects (Segura et al., 2012; Liu et al., 2016; Huang et al., 2019). Current common multi‐locus models (FarmCPU and BLINK) differ in that each SNP has only a fixed effect; thus, epistasis is additive with no interaction (first‐order effects) (Segura et al., 2012; Liu et al., 2016; Huang et al., 2019). The BSLMM is a hybrid form of LMM and Bayesian variable regression that addresses the sparsity and high‐dimensional nature of SNP data sets (Zhou et al., 2013; Gompert et al., 2017). Specifically, BSLMM considers the relatedness among individuals based on kinship and population structure similar to the LMM and estimates the proportion of phenotypic variation explained by the available genotypes (PVE). From the PVE, BSLMM estimates the combinatory influence of SNPs with polygenic (α; second‐order effect) or sparse (β; first‐order effect) effects. The proportion of PVE explained by sparse effects (unique marker first‐order effects independent of polygenic second‐order effects) is denoted as the proportion of genetic variance explained (PGE), which has an approximation (Rho) on a scale of zero to one. Rho indicates the degree to which the proposed genetic architecture is more polygenic (0) or oligogenic (1), and is accompanied by an estimated number of major effect loci (N.Gamma) and the proportion of SNPs with nonzero effects (Pi) that are either major‐effect loci or contribute to the polygenic influence.

Additionally, GEMMA returns each marker's posterior inclusion probability (PIP or γ in the BSLMM's specific terms), providing evidence for association with the phenotype comparable in interpretation to hypothesis testing in the LMM framework. In Bayesian approaches, however, thresholds that are not biologically relevant or important for the downstream use of results are unnecessary; for instance, in LMM frameworks, *P* values indicate “statistical significance.” In the context of GWA of SNP data, *P* values are the probability of observing the effect of a polymorphic state at least as extreme as the observed results, given an assumedly truthful null hypothesis of zero effect. By contrast, in a Bayesian approach for GWAS, we often use a combination of Bayes factors: the probability of a polymorphic effect being nonzero relative to the probability of a polymorphic effect being zero integrated over a prior or posterior inclusion probability (PIP), indicating the proportion of times a polymorphism's effect is estimated to have a nonzero effect during Markov chain Monte Carlo (MCMC) sampling. Within BSLMM, the PIP is often used similarly to *P* values; however, they do not have similar properties or caveats associated with multiple testing corrections (Gelman and Tuerlinckx, 2000). For example, in instances including normal or roughly homoscedastic data and a normal prior centered at zero, the estimated Bayesian effect interval is always more likely to include zero than the frequentist interval, making it far less likely to result in claims with confidence (type S error). The type S error rates for Bayesian approaches are therefore extremely low compared with frequentist approaches, making multiple testing corrections unnecessary in a BSLMM (Gelman and Tuerlinckx, 2000). The PIP is simply an indicator of how often a polymorphic state is associated with a given trait in the variety of genomic backgrounds present in the study.

Comparisons between LMMs and BSLMMs demonstrate that the latter have increased genomic prediction accuracy and estimation of PVE (Zhou et al., 2013); however, Gompert et al. (2017) made one of the few attempts to evaluate BSLMMs under simulated demographic histories, genetic architectures, and sampling designs to consider the estimations of PVE, causative effects of loci, and the number of polygenic SNPs (Gompert et al., 2017; Lind et al., 2018). To our knowledge, no published work has yet attempted to evaluate proposed genetic architectures among BSLMM and LMM approaches based on prior biological insight into a phenotype's genetic architecture and regulatory control. Additional analyses incorporating prior biological insight allow for examining proposed architectures in relevant contexts. Furthermore, we assert the terminology “proposed genetic architecture,” because the results of these analyses are statistical in that they produce a hypothesis that requires mechanistic validation.

In addition to GWAS with diverse germplasm panels, in which modeling accounts for linkage and shared allelic history, biparental experimental crosses have been used in several species to explore the genetic architecture of traits; for example, an experimental cross aims to maximize allelic diversity with crosses of phenotypically and genotypically distinct parents to allow for the mapping of quantitative trait loci (QTLs) in the resultant offspring (Gilmour, 2007; Myles et al., 2009). However, variants found in experimental crosses are not always translatable to observed variation in the general population and can only incorporate a few parental lines (Myles et al., 2009; Huang et al., 2015). In sunflower (*Helianthus annuus* L.), one QTL for lemon ray flower color (derived from carotenoids) was identified on chromosome 11 during an experimental cross of an oilseed maintainer and a confectionary sunflower (Yue et al., 2008); however, this QTL was not recovered in analyses of total carotenoid content using an association mapping panel of 288 diverse genotypes, and a new QTL on chromosome 15 was identified (Dowell et al., 2019a). To prioritize SNPs for functional analyses, we therefore need ways to break up linkage groups accounting for shared allelic history and supply detailed analyses of large SNP data sets.

Biosynthetic pathways and metabolite variation are prime examples of systems that could be used to compare proposed genetic architectures because variation in the observed output of a metabolite or metabolite class is primarily due to variation in both enzymatic efficiency and regulatory control (Badouin et al., 2017; Leong and Last, 2017; Dowell and Mason, 2020). Among metabolites, carotenoids are the second most abundant naturally occurring pigments on earth, comprising over 1000 related metabolites (Nisar et al., 2015). These compounds function as key metabolites for photoprotection via non‐photochemical quenching and other diverse functions, including visual signaling to pollinators or fruit dispersers. While most land plants possess carotenoids essential to the xanthophyll cycle, the carotenoid diversity of sunflower is yet to undergo an extensive assessment. Among sunflower petals, Kishimoto et al. (2007) identified several carotenoids underlying color in four varieties (Sunrich Orange, Sonia, Sunrich Lemon, and Valentine), such that increases in carotenoid concentration were associated with a transition from yellow to orange color; however, there were no changes in the diversity of carotenoids related to the color changes (violaxanthin, (9Z)‐violaxanthin, lutein, antheraxanthin, and zeaxanthin). In addition, β‐carotene is a commonly identified carotenoid in sunflower seed oil (Fromm et al., 2012). Alongside carotenoids essential to primary metabolism, several other enzymes along the carotenoid pathway lead to the production of non‐carotenoid specialized metabolites (Liang et al., 2011; Al‐Babili and Bouwmeester, 2015; Nisar et al., 2015; Pott et al., 2019; Dowell et al., 2019a). Knowledge of how the carotenoid pathway is regulated in eudicots, combined with previously identified QTLs in cultivated *H. annuus* and the genomic locations of enzymes within the carotenoid pathway, allows for the interpretation of GWAS results within a biological context. In addition, disentangling associations within and outside the carotenoid pathway may lead to further insights.

A candidate pathway association (CPA) is a specific case of GWAS used across the literature in a variety of styles that subsets genome‐wide SNPs to those found in collections of genes of interest, sets of transcription factors, or biochemical pathways (Payton et al., 2001; Wilkening et al., 2009; Fernàndez‐Castillo et al., 2012; Xu et al., 2014; Lin et al., 2018). However, this analytical approach has become less favorable than leveraging genome‐wide data, even though polymorphisms with low allele frequencies may necessitate candidate approaches (Wilkening et al., 2009). Furthermore, there is little consensus in the use of the term CPA to describe the scale of the analysis among studies, although the term loosely describes analyzing an a priori–selected set of genetic variants with an assumed or hypothesized interaction. However, concisely defining analytical approaches is vital to facilitate method development and improvement. Here, we propose CPA as a complementary approach to GWAS to explore how enzymatic variation in a canonical biosynthetic pathway can contribute to observed variation in a particular phenotype. In the special case of metabolism, many minor variants among enzymes within a biosynthetic pathway can contribute to observed metabolite variation; however, major transcription factor variation may obscure the detection of variation in enzyme ability and the resultant effects on metabolites or classes of metabolites through the traditional GWA approach. Furthermore, we position CPA as a tool to explore the relationship between observed metabolite variation and variation among biosynthetic enzymes.

The objectives of this study are (1) to determine whether we can leverage the low memory requirements of the LMM with the complex genetic architecture assessment of the BSLMM to provide a post‐GWA investigation technique that can be readily integrated into the many GWAS pipelines currently in use; (2) to assess the viability of CPA; and (3) to assess the ability of these methods to recapitulate previously identified QTLs in an association mapping panel of cultivated sunflower varieties.

## METHODS

### Association mapping panel and SNP set

We selected an existing association mapping panel of cultivated sunflower varieties known as the sunflower association mapping (SAM) panel (Mandel et al., 2013). The SAM panel consists of 288 inbred lines, which together capture approximately 90% of the allelic diversity among cultivated sunflower varieties present within the germplasm repositories of the United States Department of Agriculture (USDA) and the French Institut National de la Recherche Agronomique (INRA) (Mandel et al., 2013). This panel has been used to map a range of phenotypes to the sunflower genome (Mandel et al., 2013; Masalia et al., 2018; Dowell et al., 2019a; Temme et al., 2020). Work performed with this panel before the publication of the sunflower pangenome (Hübner et al., 2019) used a low‐density SNP map (5788 SNPs; Mandel et al., 2013), including work mapping petal carotenoid content (Dowell et al., 2019a). More recently, as part of the development of the sunflower pangenome, each genotype of this panel has been subjected to whole‐genome shotgun resequencing (Hübner et al., 2019). From the resequencing data, Todesco et al. (2020) developed a high‐density map of 1.4 million SNPs across all 17 chromosomes of *H. annuus* with a minor allele frequency <5% and heterozygosity >10%, which we used for this analysis.

### Carotenoid content

**Figure 1:**
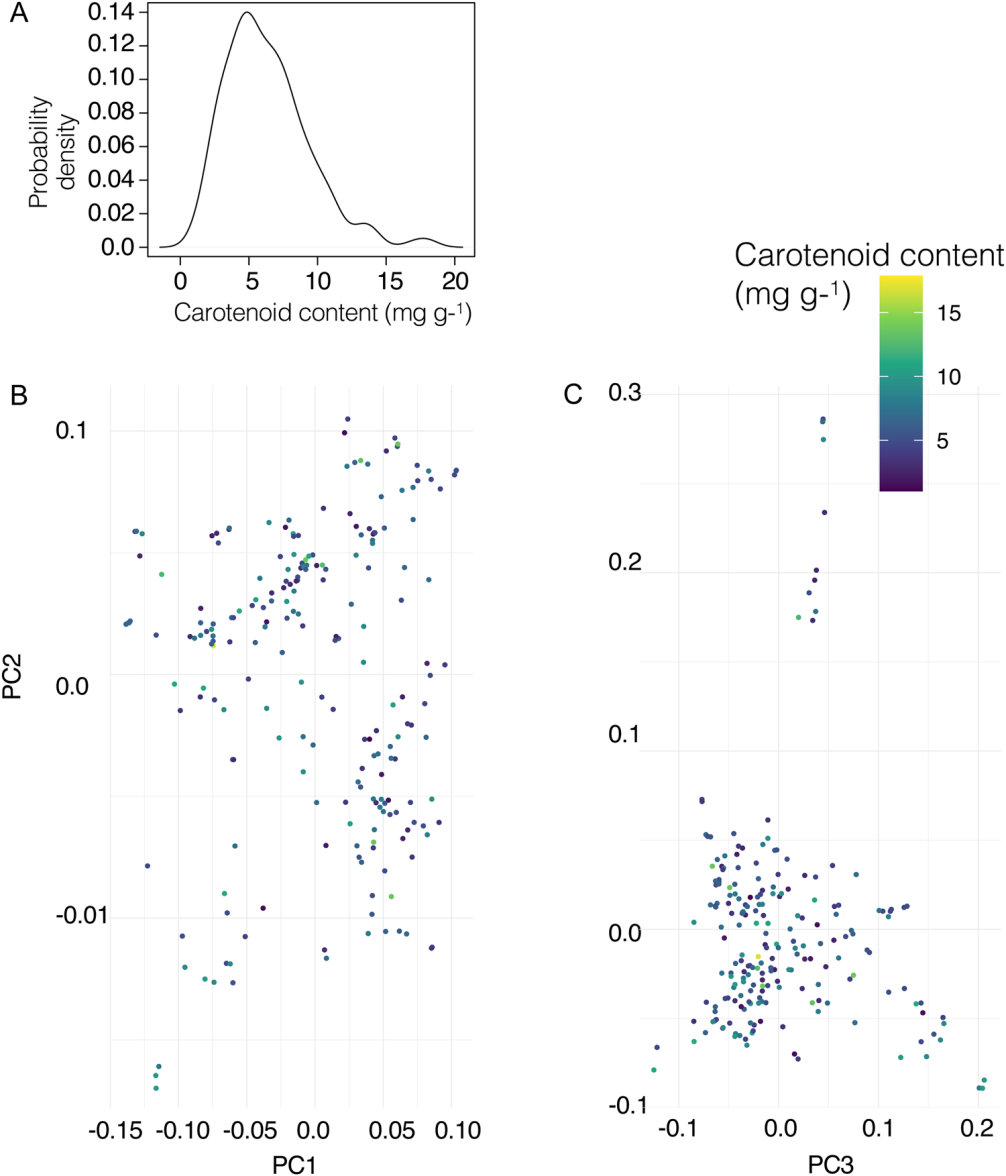
Variation in carotenoid content across the sunflower association mapping (SAM) panel. (A) Density plot of the distribution of carotenoid content observations among sunflower lines. (B, C) Population structure estimated from a set of over 24,000 independent single‐nucleotide polymorphisms (SNPs) using a sliding window approach (Tajima's *D*′ < 0.8) via a principal component analysis. Individuals are colored based on their relative carotenoid content, from high in yellow to low in dark blue.

Phenotypic data were retrieved from the Dryad Digital Repository entry (https://doi.org/10.5061/dryad.26t7cs8; Dowell et al., 2019b), and methods for phenotypic data collection are described in Dowell et al. (2019a) (Figure 1). In the original study, three to four replicate plants per genotype were grown under environmentally controlled conditions at the University of Georgia Plant Biology Greenhouses in Athens, Georgia, USA. At flowering, three to five ray floret petals were snap‐frozen and stored at −80°C. To quantify the petal carotenoid content, pigments were extracted from the petals using methanol, and their absorbance at 436 nm was recorded using visible spectrometry (Barrell et al., 2010). The absorbance values were converted to mg g^−1^ equivalents of β‐carotene (CAS #7235‐40‐7; Sigma‐Aldrich, St. Louis, Missouri, USA).

### Linear mixed model GWA

We performed all analyses using the GWASvsCPAS pipeline (Dowell, 2021), with all association analyses performed using GEMMA version 0.98.1 (Zhou and Stephens, 2012) (Appendix S1; see Supporting Information with this article). We initially performed an LMM with kinship and population structure corrections, previously calculated in Temme et al. (2020). Briefly, GEMMA was used to estimate kinship. By contrast, the population structure was estimated with principal component analysis from a set of over 24,000 SNPs independently identified using a sliding window approach (Tajima's *D*′ < 0.8), where the first four principal components were used as estimates of population structure (Temme et al., 2020). To correct the significance thresholds for multiple comparisons, we used a Gao correction, for which the 20,562 multi‐SNP haplotype blocks identified comprise the number of independent tests (Gao et al., 2008, 2009; Temme et al., 2020). While not as extreme as a Bonferroni multiple‐testing correction, this type of correction remains a highly conservative significance threshold dependent on the accuracy of the genome assembly that preserves reasonable statistical power (Temme et al., 2020). For analysis in a BSLMM framework, we further parsed the LMM results into three data sets based on the top 1% of the lowest *P* values (“1% *P* values SNP set”), the highest 1% of β‐effect sizes (“1% effect size SNP set”), and the union of both SNP data sets (“Combination SNP set”). In combination, we attempted to incorporate the multi‐locus models FarmCPU and BLINK; however, the memory requirements quickly exhausted a Mac M1 CPU processor with 16 GB RAM and eight cores. We therefore performed a subsequent analysis of the SNP subsets using FarmCPU and BLINK.

### Bayesian sparse linear mixed model GWA

We used the following methods to analyze the genome‐wide SNP data set and SNP subsets taken from our LMM results. For kinship and population structure, we used the same estimates as in our LMM (Temme et al., 2020). We set most parameters to default values for ease of assessment, changing only three: burn‐in to 500,000, MCMC steps to 5,000,000, and recording pace to 100. Although priors can be set for all parameters, we chose default parameters to ensure the transferability of our approach. We ran each analysis 10 times and reported the median values. From the resulting analyses, we expressed the effect of each SNP as the absolute value of β × γ, where β is the main effect of the SNP and γ is the PIP of the SNP's association with the variation of the phenotype in the presence of the population structure. The effect we report varies based on the unique effect of each SNP (β) and the polygenic background of second‐order effects (γ). While thresholds in Bayesian approaches are arbitrary, we used the criteria of a PIP of 10% or higher and having a highest posterior density interval (HPDI) not overlapping zero to select which SNPs to investigate further and classify as major effect loci. The value of 10% indicates that in at least 10% of the MCMC samplings, these SNPs exhibited nonzero effects. A PIP points toward a SNP's association, and an HPDI that does not cross zero suggests a common directional effect in various genomic backgrounds.

### Candidate pathway association

**Figure 2:**
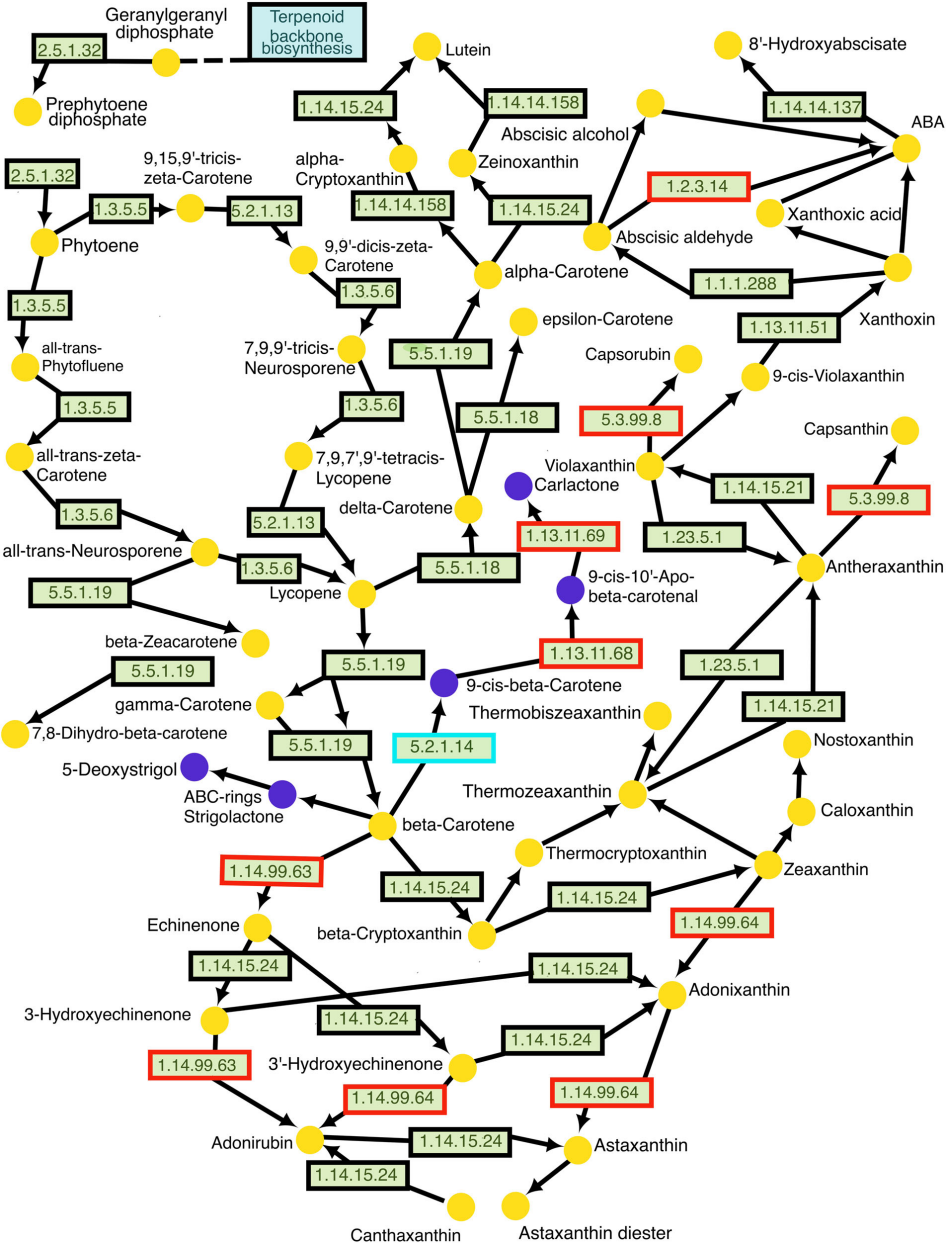
Carotenoid pathway based on the *Helianthus annuus* (HA412HO) genome annotation. Green rectangles represent biosynthetic enzymes in the carotenoid pathway present in the annotation of the *Helianthus annuus* genome (Ha412HO version 2.0). Red outlined enzymes indicate enzyme sequences that have no polymorphisms within our association mapping population. The blue outline indicates a β‐carotene isomerase associated with carotenoid content identified using candidate pathway association with a Bayesian sparse linear mixed model using SNPs found in biosynthetic enzymes of the carotenoid pathway. Circles indicate metabolites, with general carotenoids colored yellow and other specialized metabolite precursors in purple.

For the CPA, we used the Kyoto Encyclopedia of Genes and Genomes (KEGG) database to curate a list of enzymes in the carotenoid biosynthetic pathway present in cultivated *H. annuus* (Figure 2) (Kanehisa and Goto, 2000). The enzyme commission numbers were compared with the annotation of the sunflower genome HA412HO version 2.0 to locate enzyme‐encoding genes across the genome (Todesco et al., 2020). Finally, we compiled a list of SNPs found among the enzyme‐encoding genes from the annotated locations and analyzed the set of carotenoid biosynthetic enzyme SNPs in both the LMM and BSLMM frameworks using the kinship, population structure, prior specifications, and convergence criteria previously detailed (Figure 2).

## RESULTS

We designed the present study to examine the variation between genomic architectures described using LMMs and BSLMMs in GWA and CPA frameworks. In addition, we report the mean and 97.5% HPDI for all hyperparameters.

### Linear mixed modeling and BSLMM of genome‐wide SNPs

**Figure 3:**
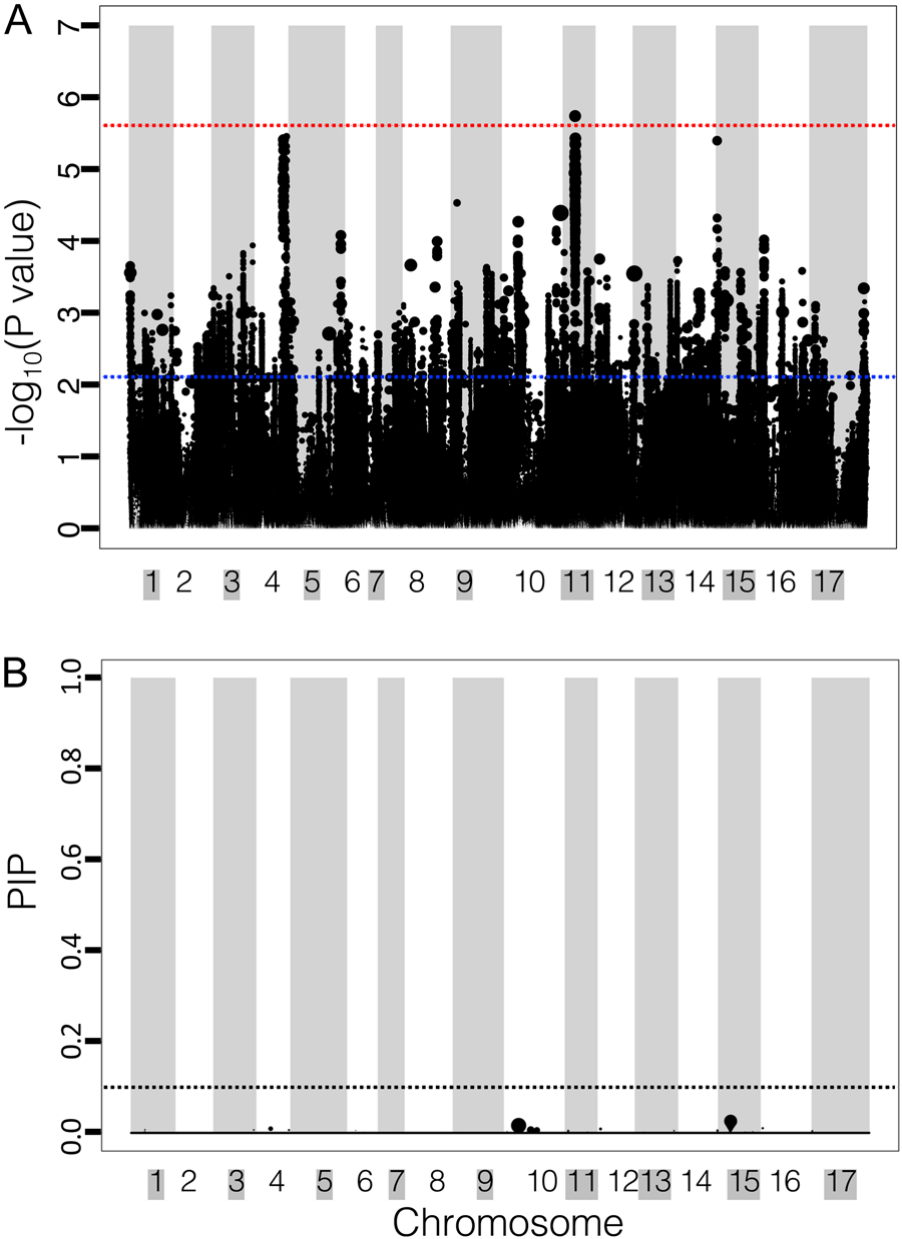
Genome‐wide association of carotenoid content in cultivated *Helianthus annuus*. (A) Linear mixed model (LMM) results. The red line indicates the more conservative Gao threshold; the blue line indicates –log_10_(0.001). (B) Bayesian sparse linear mixed model (BSLMMM) results. Dotted line indicates a posterior inclusion probability (PIP) of 0.1. In both figures, the size of the circle is relative to the effect size of the SNP. The width of the column is relative to the number of SNPs within that region. Chromosome colors (white or gray) are matched to the background color of the chromosome name (white or gray).

Among the genome‐wide SNPs analyzed in the LMM framework, only one SNP was significantly associated (Ha412HOChr11:41892223) with the carotenoid content, with a relative effect size of 16%. This SNP is centered in a 147.263‐kbp region on chromosome 11 (Figure 3A, Appendix S1). Forty‐six other SNPs are located in this region, which contains a single gene (Ha412HOChr11g0480931) with a putative function relating to DNA‐binding transcription factor activity (gene ontology terms: GO:0003700, GO:0006355) (Appendix S1).

**Figure 4:**
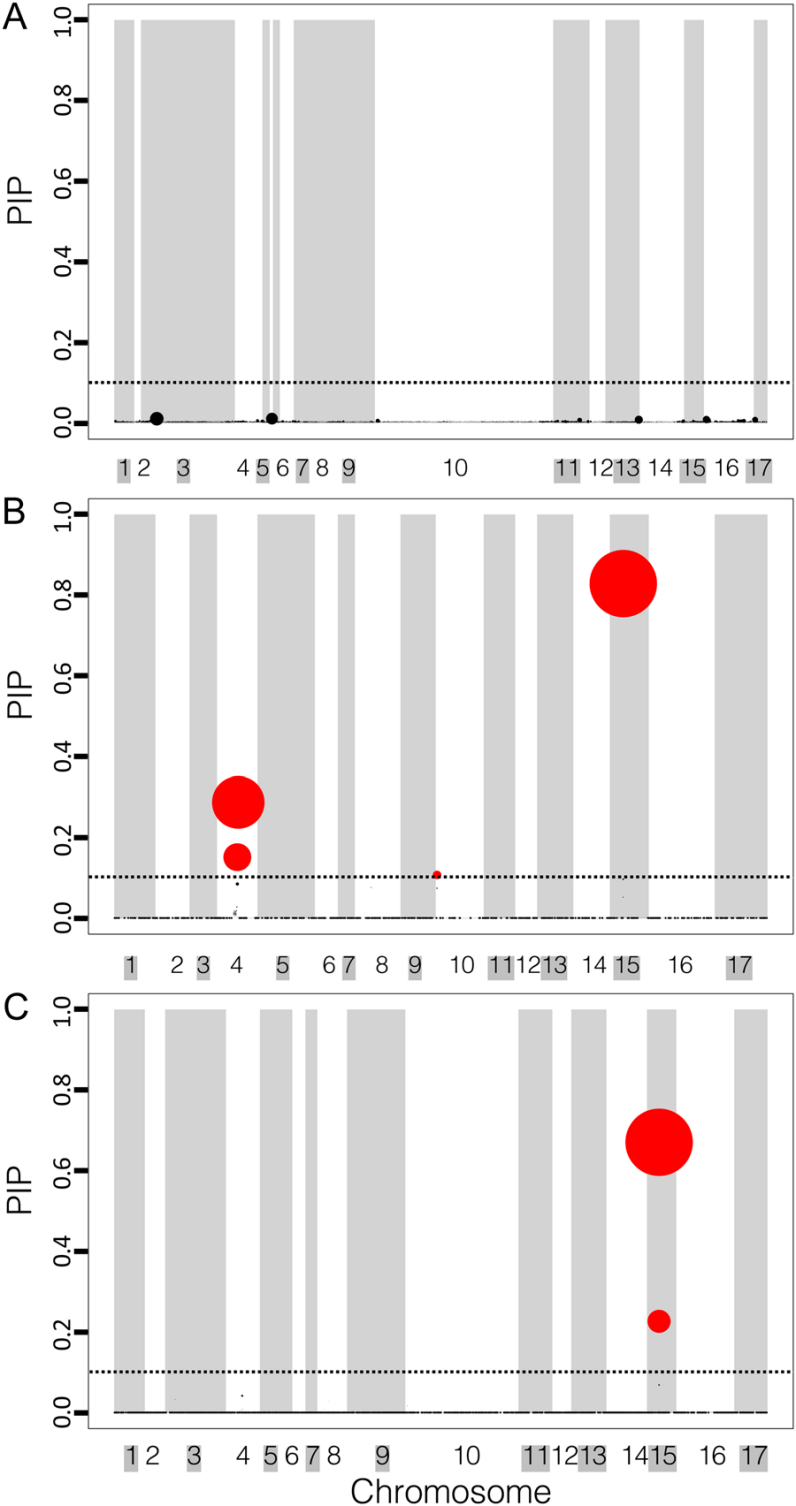
BSLMM analysis of genome‐wide SNP subsets based on LMM results of carotenoid content in cultivated *Helianthus annuus*. (A) The top 1% of *P* value SNPs. (B) The top 1% of effect size SNPs. (C) The combination of SNPs from A and B. Dotted lines indicate a posterior inclusion probability (PIP) of 0.1, with SNPs with a PIP > 0.1 in red. The size of the circle is relative to the effect size of the SNP. The width of the column is relative to the number of SNPs within that region. Chromosome colors (white or gray) are matched to the background color of the chromosome name (white or gray).

The BSLMM hyperparameters indicated a wide range of potential architectures. The Rho value (0.62; HPDI = 0.20–0.96) skewed toward zero (Appendix S2), indicating a likely oligogenic architecture (Zhou et al., 2013). The estimates of PVE were lower in the BSLMM (47%; HPDI = 29–64%), with estimates of 58% (HPDI = 33–94%) of the PVE explained by large‐effect SNPs (PGE; the proportion of genetic variance explained by major‐effect loci) (Tables 1 and 2; Appendix S2). The Pi estimates of the SNPs indicated nonzero effects (0.029%; HPDI = 0.007–0.133%), and approximately three of the SNPs assessed should have major effects (N.gamma = 3; HPDI = 1–19; Table 2, Appendix S2). Among the SNPs with a PIP > 0.01, 78% were in a 629.867‐kbp region of chromosome 15 (32229872–32859738 bp), which contained a putative photosystem I (PSI) assembly factor (*PHOTOSYSTEM 1 ASSEMBLY 3* [*PSA3*]; Ha412HOChr15g0707801) and a putative monodehydroascorbate reductase gene (Ha412HOChr15g0707991) (Figure 4B, Appendix S1), which is vital for the production of ascorbate (vitamin C) (Cazzonelli and Pogson, 2010). This region had a relative effect size of 16% and a PIP of 0.03, leading to a low sparse effect of 0.02, indicating this variant appeared a low number of times in the MCMC sampling with a nonzero effect.

**Table 1:** Narrow sense (*h* ^2^_SNP_) heritability in LMMs and a comparable measure of the percentage of phenotypic variance explained by genotype (PVE) estimates across BSLMMs. Values in parentheses are a 97.5% confidence interval for the LMMs and the 2.5–97.5% highest posterior density interval (HPDI) for BSLMMs.

*h* ^2^ estimates	GWA	CPA
Linear mixed model	56% (34–78%)	56% (45–67%)
Bayesian sparse model	47% (29–64%)	39% (15–60%)
1% *P* values SNP set	28% (6–49%)	
1% effect sizes SNP set	38% (20–55%)	
Combination SNP set	33% (17–51%)	

*Note*: CPA = candidate pathway analysis; GWA = genome‐wide association.

**Table 2:** Mean hyperparameter estimates across BSLMMs. Values in parentheses include the 2.5–97.5% highest posterior density interval (HPDI).

Analyses	PGE	Rho	Pi	N.Gamma
CPA	34% (7.5–82%)	0.39 (0.04–0.87)	10% (0.002–0.41%)	69.1 (1–279)
GWA	61% (34–94%)	0.65 (0.22–0.98)	2.8e^–4%^ (7.2e^–7^–1e^–5^%)	4.1 (3–14)
1% *P* values SNP set	19% (0–74%)	0.31 (0.01–0.88)	0.27% (0.0073–1.3%)	39 (0–188)
1% effect sizes SNP set	75% (41–98%)	0.81 (0.31–0.99)	0.027% (0.0073–0.091%)	4.37 (1–12)
Combination SNP set	70% (36–98%)	0.70 (0.24–0.99)	0.016% (0.0036–0.01%)	4.7 (1–20)

*Note*: CPA = candidate pathway analysis; GWA = genome‐wide association; N.Gamma = number of loci with major effects, an approximation of Pi; PGE = percent of the percent of phenotypic variance explained by genotypes (PVE) explained by major effect loci; Pi = proportion of loci with nonzero effects; Rho = an approximation of PGE.

### Bayesian sparse linear mixed model of SNP subsets

Among the SNP subsets, we did not identify any SNPs within any genes encoding carotenoid pathway enzymes. The estimates of PVE were broadly comparable among the SNP subsets, with the highest estimated PVE observed for the “1% effect size SNP set” and the lowest for the “1% *P* values SNP set” (Table 1, Appendix S2). The “1% effect size” and “Combination SNP” sets indicated an oligogenic architecture, while the “1% *P* values SNP set” indicated a polygenic architecture (Table 2, Appendix S2). A similar pattern occurred for the percent of PVE explained by sparse effects (major‐effect loci or first‐order effects) (PGE) in the “1% *P* value SNP set,” which explained a small amount of the PVE (PGE = 19%; HPDI = 0–74%), and the “1% effect size SNP” and “Combination SNP” sets, which explained over half of the PVE (PGE = 75% [HPDI = 41–98%] and 70% [HPDI = 36–98%], respectively; Appendix S2). We observed similar patterns for N.gamma and Pi, for which the effect size and combination SNPs both had similar estimates to the proportion of SNPs with nonzero effects and the number of major‐effect loci (Table 2, Appendix S2). By contrast, the “1% *P* values SNP set” had a higher count of SNPs with estimated nonzero effects (Table 2).

Among the major‐effect loci, both the “Combination SNP” and “1% effect size SNP” sets indicated one SNP associated with carotenoid content located on chromosome 15 in the coding sequence of the same gene, putatively encoding PSA3 (Ha412HOChr15g0707801). This specific SNP (Ha412HOChr15:32353233) may contribute to a missense amino acid change from lysine to arginine, while any single insertion or deletion at this locus may produce a premature stop or new start codon. The resequencing depth of the population ranged from 5–25× (Hübner et al., 2019), so a higher sequencing depth is needed to fully determine the sequence variation to rule out the possibility of small structural variants (Dierckxsens et al., 2021). The BSLMM of the global data set also suggested the presence of a putative monodehydroascorbate reductase (Ha412HOChr15g0707991) in the same linkage group (Figures 3 and 4B, Appendix S1). The “1% effect size SNP set” implicated SNPs on both chromosome 10 and chromosome 4 associated with carotenoid content (Figure 4B). Although almost all of the SNPs identified using the “1% *P* values SNP set” had a nonzero effect, we did not find any major‐effect loci in the SNP set (Figure 4A). Both multi‐locus models generally agreed with the results of the BSLMMs of SNP subsets, further verifying the major findings of the BSLMM models.

### Candidate pathway association

**Figure 5:**
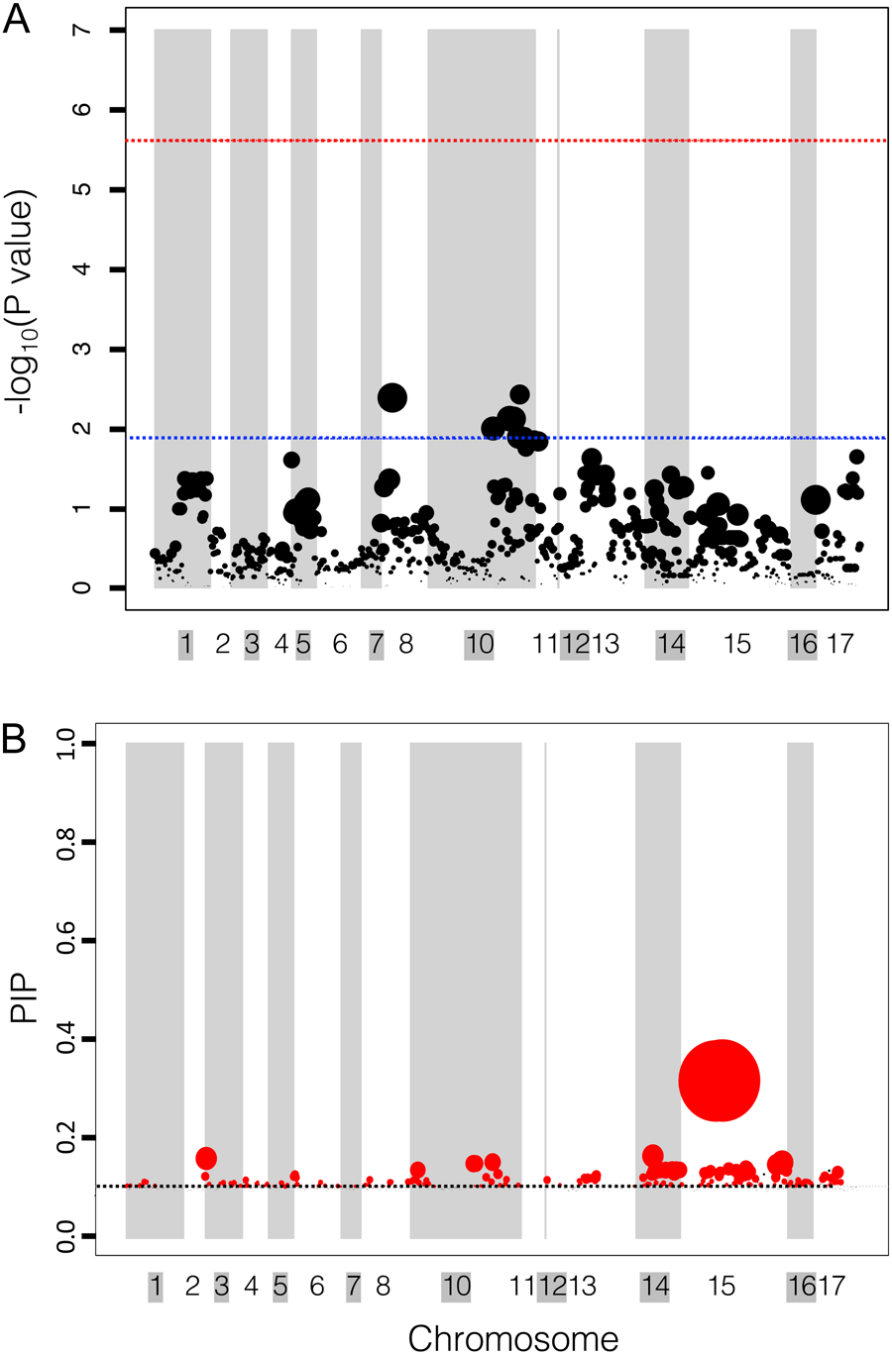
LMM and BSLMM analyses of SNPs within the carotenoid pathway of cultivated *Helianthus annuus*. (A) LMM results. Chromosome 9 is missing, as no SNPs were present on this chromosome. The red line indicates the more conservative Gao threshold; the blue line indicates –log_10_(0.001). (B) BSLMM results. The dotted lines indicate a posterior inclusion probability (PIP) of 0.1. The size of the circle is relative to the effect size of the SNP, with SNPs with a PIP > 0.1 in red. The width of the column is relative to the number of SNPs within that region. Chromosome colors (white or gray) are matched to the background color of the chromosome name (white or gray).

In the KEGG carotenoid pathway, 16 out of 21 unique enzyme‐encoding genes displayed polymorphisms within our data set (Figure 2). We identified a total of 687 SNPs among the carotenoid‐biosynthesis genes annotated in the sunflower genome (Todesco et al., 2020). The LMM did not reveal any SNPs above the Gao threshold set by the total number of SNPs in the global data set (Figure 5A).

The BSLMM of the carotenoid pathway SNPs indicated a comparable PVE between the “1% effect size SNP” and “Combination SNP” sets; however, the Rho (0.39 [HPD = 0.04–0.87]) was skewed right (Appendix S2), indicating a polygenic architecture (Zhou et al., 2013). All of the SNPs had nonzero effects, and the top two SNPs with the highest sparse effects (both 0.12) are located in a β‐carotene isomerase gene (Ha412HOChr15g0697271) on chromosome 15 (6724573–6729321 bp) (Figures 2 and 5B). Specifically, the Ha412HOChr15:6728659 SNP is in the intron between the second and third exon, and the Ha412HOChr15:6726790 SNP is in the first position of a stop codon on the third exon. β‐carotene isomerase specifically converts β‐carotene to 9‐*cis*‐β‐carotene, which is ultimately converted to carlactone, a strigolactone‐like plant hormone. From β‐carotene, there are four potential biosynthetic paths (Figure 2). Two of the four paths direct β‐carotene toward the production of compounds participating in the photoprotective xanthophyll cycle, although in one of these, the conversion of β‐carotene to echinenone and several other enzymatic steps show no polymorphisms in our mapping population (Figure 2). The other two paths generate strigolactones and carlactone.

## DISCUSSION

### Conflicting genetic architectures for petal carotenoid content

It is a frequent practice to leverage SNP data in an LMM framework to explore the genetic architecture of traits and prioritize SNPs for functional analyses across plants, animals, and microbes; however, there has been little critical evaluation of alternative methods to the LMM that focus on the potentially conflicting genetic architectures that alternative methods may propose. Here, we compare the genetic architectures proposed by GWA and CPA in Bayesian sparse and traditional LMM frameworks, supply a post‐GWA examination approach using BSLMMs, and explore the architectures proposed for petal carotenoid content based on known biosynthetic enzymes in cultivated *H. annuus*.

There are discrepancies among the proposed architectures between the LMMs and BSLMMs of genome‐wide SNPs. The LMM identified a SNP on chromosome 11 related to an unknown transcription factor not found in any other analysis; however, a linkage group of approximately 10 cM from this identified SNP's linkage group has previously been linked to lemon ray flower color in an experimental cross of cultivated *H. annuus* (Yue et al., 2008). In the BSLMM of genome‐wide SNPs, the SNP with the highest effect was on chromosome 15, and analyses of the “1% effect size SNP set” and “Combination SNP set” both corroborated this result. Chromosome 15 was also previously linked to carotenoid content in this association mapping panel, alongside floral ultraviolet (UV) pigmentation in a GWAS of *H. annuus* and *H. petiolaris* subsp. *petiolaris* (Nutt.) Anashch. (Dowell et al., 2019a; Todesco et al., 2022). Among the three SNP sets, the “1% effect size SNP set” proposed two new potential QTLs for petal carotenoid content. Interestingly, none of the variants identified from the GWAS across all SNP sets implicated any biosynthetic enzymes in the carotenoid pathway. Furthermore, an assessment of these two potential QTLs revealed they were not linked to any known specialized metabolite biosynthetic genes, although these regions did include many genes associated with antioxidant activity and transcriptional regulation (Appendix S2).

Interestingly, the CPA revealed conflicting proposed architectures with the GWA. The LMM results of the carotenoid pathway SNPs did not identify any SNPs above the significant or suggestive thresholds, and indicated that none of the individual variants within the biosynthetic enzymes significantly impacted the carotenoid content. However, the same observed total class concentration (e.g., “total carotenoids”) may arise from several combinations of unique compounds, obscuring the detection of within‐pathway control. Alternatively, the BSLMMs generated more nuance in describing the variation within and among biosynthetic enzymes, in that all biosynthetic enzymes had a nonzero effect on the observed carotenoid content. The nonzero effects across the SNPs within genes encoding biosynthetic enzymes indicate that these variants may function in a combinatory fashion to produce similar amounts of total carotenoids. Among the carotenoid pathway enzyme genes, a β‐carotene isomerase (Ha412HOChr15g0697271) had the largest effect on observed petal carotenoid content. Future work leveraging CPA should examine the relationship between compound class totals, individual compounds, and the impact of detecting associated SNPs, particularly emphasizing the influences of pathway structure and reaction types involved.

### Biological insights into carotenoid biosynthesis through BSLMMs

While originally considered secondary or unnecessary for growth or reproduction, specialized metabolic pathways are strongly integrated with primary metabolism, with many nexus points between primary and specialized metabolisms across various biosynthetic pathways. In the carotenoid pathway, core photosynthetic pigments classically considered primary metabolites mediate abiotic stress responses (e.g., the photoprotective xanthophyll cycle) and are closely integrated to the production of key compounds more commonly considered secondary for their roles in biotic interactions (e.g., strigolactones mediating mycorrhizal symbiosis and parasitic plant resistance).

In *Helianthus* L., Todesco et al. (2022) implicated the same 629.867‐kbp region of chromosome 15 (32229872–32859738b) that we identified in a GWAS of floral UV pigmentation patterns and flavonol accumulation in the petals of wild *H. annuus* and *H. petiolaris* subsp. *petiolaris* plants. Within this linkage group, the transcription factor MYB111 impacts UV pigmentation and flavonol accumulation in the petals of *H. annuus* and *Arabidopsis thaliana* (L.) Heynh. (Todesco et al., 2022). *MYB111* and other *MYB* genes are coexpressed with various flavonoid, anthocyanin, carotenoid, and vitamin C biosynthetic pathway genes in *Capsicum chinense* Jacq., suggesting a regulatory module controlling this cluster (Islam et al., 2021). In addition to MYB111, SNPs in the haplotype block of the sunflower chromosome 15 are also associated with a putative monodehydroascorbate reductase (Ha412HOChr15g0707991), which reduces radical ascorbate into l‐ascorbate (vitamin C). In plants, monodehydroascorbate reductase forms a core component of the glutathione‐ascorbate cycle, one of the major antioxidant systems in plants (Cazzonelli and Pogson, 2010). Reactive chemical species are produced in many instances in which carotenoids are deployed to ameliorate abiotic stress conditions, such as through nonphotochemical quenching or combating pests and pathogens with specialized metabolites (Roseland and Grosz, 1997; Costa França et al., 2000; Nisar et al., 2015; Ameye et al., 2020). Violaxanthin, antheraxanthin, and zeaxanthin are major carotenoids responsible for the nonphotochemical quenching of excess light energy, and their relative quantities are linked to ascorbate levels (Cazzonelli and Pogson, 2010). Specifically, the de‐epoxidation of violaxanthin to antheraxanthin and zeaxanthin in the xanthophyll cycles requires ascorbate (Rockholm and Yamamoto, 1996). In addition, increases in the concentration of ascorbate increase the stability of β‐carotene, both over time and in the presence of increasing light intensity and temperatures ex vivo (Morais et al., 2002). Alternatively, the monodehydroascorbate reductase is an oxidoreductase, as is xanthoxin dehydrogenase in the carotenoid pathway. Studies show oxidoreductases can be substrate‐ and product‐promiscuous, and are implicated in several specialized metabolic processes (Leong and Last, 2017). To explore the links between ascorbate and carotenoid production, promiscuous enzymes may be vital in connecting the coregulation or coordinated production of distant pathways.

In petals, carotenoids and other petal pigments are accumulated in chromoplasts, plastids derived from chloroplasts. Provided that both plastids leverage carotenoids in some manner and lipid membranes are necessary for accumulation and physiological functions, they may use similar molecular machinery. In Todesco et al. (2022), this same locus is implicated in a genome environment association (GEA) of average temperature in *H. annuus* and *H. petiolaris* subsp. *petiolaris* (Todesco et al., 2022). In the context of photosynthesis, a SNP (Ha412HOChr15:32353233) in the coding sequence of the putative PSI assembly factor PSA3 (Ha412HOChr15g0707801) may have implications for the relationship between photosystem health, carotenoid investment, and the integrated association with temperature adaptation in a potential *cis*‐regulatory module on chromosome 15. The PSI super‐complex includes 16 protein subunits and over 200 prosthetic groups, primarily light‐harvesting pigments, specifically carotenoids. PSA3 is a conserved protein in green photosynthetic eukaryotes on the stromal face of the thylakoid membrane, promoting PSI accumulation alongside assembly factor tetratricopeptide repeat protein PYG7 (Shen et al., 2017). Loss‐of‐function mutations in PSA3 in maize (*Zea mays* L.) and *A. thaliana* result in the loss of PSI formation, even though there is no impact on PSI subunit biosynthesis (Shen et al., 2017). While *psa3* mutants exhibit pale green leaves (Shen et al., 2017), we are not aware of any studies assessing the impacts on other tissues where carotenoid accumulation is important, such as petals. In developing mechanistic hypotheses, structural predictions suggest that PSA3 binds basic peptides and is sensitive to the oxidative state of cysteine pairs flanking its predicted peptide‐binding groove (Shen et al., 2017). In the present study, the SNP identified in the sunflower PSA3 (Ha412HOChr15:32353233) may contribute to an amino acid substitution of lysine for arginine. Lysine and arginine are both basic amino acids, and studies of the interactions of these side chains with lipid membranes show similar free energy barriers when crossing membranes (Li et al., 2013); however, in a charged state, lysine becomes deprotonated in lipid membranes. By contrast, arginine can maintain its charge, leading to enhanced interfacial binding and membrane perturbations, including trans‐membrane pore formation, which may lead to dehydration or membrane deformations (Li et al., 2013). Ascorbate is essential to the stability of β‐carotene, so ascorbate levels may alter the impact of amino acid substitutions on PSA3 function. Further work should assess the mechanistic links between the membrane implantation of PSA3, ascorbate levels, and various substitutions of lysine for arginine and PSI accumulation, alongside the impacts on other plastids such as chromoplasts. The importance of links between PSA3, ascorbate, β‐carotene, and flavonol accumulation suggests that the chromosome 15 locus may form a *cis*‐regulatory module influencing multiple primary and specialized metabolic pathways essential to plant physiology and petal pigmentation. Further study of the impacts of MYB111 on genes in the chromosome 15 linkage group may provide insight into the regulation of the phenotypic integration of physiology and floral pigmentation.

In a separate analysis of cross‐organ gene expression, the β‐carotene isomerase (Ha412HOChr15g0697271) identified in our CPA shows similar constitutive expression patterns in the petals, mature leaves, and roots (Badouin et al., 2017). Although the highest expression of β‐carotene isomerase occurs within stamens and disc‐ovaries (Bing et al., 2019), ray flowers accumulate 2–3× more carotenoids (Fambrini and Pugliesi, 2019). In addition, when exposed to many essential plant hormones, expression patterns of β‐carotene isomerases stay consistent between roots and leaves (Bing et al., 2019). While gene expression does not perfectly correlate with protein abundance, our CPA results suggest that enzyme variation contributes to the observed constitutive variation in the carotenoid content of the petals, and should be followed by validation. As petals in sunflower are ligules (a fused strap‐like fused corolla, which like all floral structures are ultimately derived from specialized leaf whorls), future work should also examine the expression patterns across vegetative and reproductive organs for shared inducible patterns of β‐carotene isomerase expression to identify potential cross‐organ regulation.

Although there are nuances to the impact of enzymatic reactions on plant growth and defense, examining nexus points in biochemical pathways may permit useful insights into the scale‐dependent nature of growth‐defense tradeoffs, from pathways to whole organisms. Given that (1) the production of carotenoids and carlactones are linked, (2) β‐carotene isomerase is constitutively expressed across several organs, and (3) there are few copies of β‐carotene isomerase genes, enzymatic variation may contribute to biologically relevant variations in the products of this pathway. β‐carotene isomerases link the production of β‐carotene to the production of strigolactone‐like compounds (carlactone) in roots, which is essential for plant mycorrhizal association and parasitic plant interactions (Al‐Babili and Bouwmeester, 2015). In sunflower, carlactones induce hyphal branching of the arbuscular mycorrhizal fungi *Gigaspora margarita* (Mori et al., 2016) while stimulating the germination of roots from parasitic plants such as *Orobanche cumana* Wallr. and *Striga hermonthica* (Delile) Benth. (Ueno et al., 2014). However, neither the transcriptome changes induced by arbuscular mycorrhizal fungi nor the results of an LMM GWAS of mycorrhizal associations in cultivated sunflower implicated β‐carotene isomerase or any of the other putative carotenoid‐influencing genes identified in the present study (Vangelisti et al., 2018; Stahlhut et al., 2021). While products of the carotenoid pathway can influence mycorrhizal relationships, other genetic markers, such as potassium ion channels and plant immunity gene variation, are the primary correlates of these associations in sunflower (Vangelisti et al., 2018; Stahlhut et al., 2021). Further integrating our findings, if the presence or absence of carlactones has a minimal impact on mycorrhizal associations in cultivated sunflower, future breeding work to combat parasitic plants may target β‐carotene isomerase to either reduce its enzyme efficiency or reduce its gene expression. In addition, carlactones require cost‐prohibitive analytical chemistry equipment to quantify properly; thus, as these pathways are linked, further work should investigate the utility of cost‐effective high‐throughput colorimetric or hyperspectral approaches to phenotype the carotenoid compound profiles of petals, leaves, or roots as useful indicators of the metabolic activity of the underlying pathway. Regardless, further work should examine the investment in β‐carotene in relation to strigolactones (carlactones), parasitic plants, and mycorrhizal associations in sunflower, focusing on the cross‐organism integration of metabolite production and gene regulation.

The inhibition of carotenoid biosynthesis is a major target for general herbicides. Inhibition may occur indirectly by inhibiting the catalysis of 4‐hydroxyphenyl pyruvate dioxygenase (HPPD) enzymes or more directly by inhibiting phytoene desaturase or diterpene biosynthesis (Chamovitz et al., 1993; Arias et al., 2005). When a plant is treated with carotenoid biosynthesis inhibitors, its carotenoid content is reduced, leading to an increase in unbound lipid radicals (Arias et al., 2005). Lipid radicals lead to lipid peroxidation by impairing the uptake of membrane lipids and fatty acids, negatively impacting the biosynthesis of chlorophyll and other membrane‐bound lipids and proteins (Arias et al., 2005). As one of the first enzymes in the carotenoid pathway, phytoene desaturase is one of the most important enzymes. While many inhibitors of phytoene desaturase have been biosynthesized as general herbicides, mutations of phytoene desaturase occurring repeatedly across green photosynthetic eukaryotes are a selectable marker of herbicide resistance (Chamovitz et al., 1993; Arias et al., 2005; Qin et al., 2007; Liu et al., 2013). While no SNPs in phytoene desaturase are implicated in this study, the other loci associated with carotenoid content that have potential links to photosynthesis may provide novel targets for herbicide resistance, specifically in sunflower.

### The utility of complementary GWA and CPA approaches

In traditional plant growth–defense theory, energetic tradeoffs are often estimated at the level of the whole organism or the level of constituent organs; however, tradeoffs may occur at much finer scales and be masked by larger tradeoffs elsewhere (van Noordwijk and de Jong, 1986). In the context of GWA, *cis*‐ and *trans*‐regulatory elements may obscure our ability to identify enzyme variants contributing to variation in an observed product due to correlation (positive or negative) or shared explained variance with regulatory elements. When using only LMM GWA approaches, we can bias ourselves toward identifying singular large‐effect loci that represent an oligogenic architecture. For a metabolite, large‐effect loci may include *cis*‐ or *trans*‐regulatory elements affecting multiple portions of a pathway or controlling aspects outside the pathway of interest with linked functionality (Rowe et al., 2008). In our case, the traditional LMM approach identified an unknown transcription factor. By contrast, BSLMM approaches using the genome‐wide SNP set or SNP subsets based on the LMM results identified an enzyme outside the carotenoid pathway with links to antioxidative systems.

While large‐effect regulatory loci may be useful for initial breeding purposes, this does not mean that genetic variation within the enzymes of a pathway does not contribute to overall variation. By taking a pathway‐centric view, we can narrow our search parameters to identify the most likely candidate enzyme variants within a pathway that should be subjected to a functional assessment, such as the β‐carotene isomerase CPA implicated by our BSLMM of carotenoid pathway SNPs. In addition, the genetic architecture of a metabolic profile, or the production of a specific metabolite, may be inherently polygenic due to the many biosynthetic steps involved; for example, to observe a given metabolite, all enzymes must be able to complete their reactions. Secondarily, variation in any enzyme may alter the observed amount of the metabolite, or net changes may produce the same amount of the metabolite. In this small example, one still must compound the complexity with *cis*‐ and *trans*‐regulatory elements changing the enzyme abundance and that of the resulting precursors or products. In addition, when considering multiple metabolites within a class, the correlation among observed metabolites can be explained by the presence or absence of enzymes and the shared physiochemical properties of the observed metabolites (Dowell and Mason, 2020). Thus, novel genetic association methods are needed to parse variation due to gene regulatory elements, presence/absence, and sequence variation. Given our understanding of the hierarchical nature of pathway regulation, the conceivable limitation on identifying enzymatic variants with traditional approaches necessitates complementary approaches to uncover the genetic architecture of complex traits, such as metabolism. Along the road to improved method development, we view the inclusion of CPA and BSLMM approaches to complement traditional LMM approaches as a necessary next step to further explore the genetic architecture of metabolism and other complex traits.

In conclusion, common GWA methods can propose alternative architectures. In the case of petal carotenoid content in cultivated *H. annuus*, our analyses complementarily recapitulated previously identified QTLs from association mapping and experimental crosses. Furthermore, CPA supplies novel insights into carotenoid content not observed among genome‐wide SNPs. Finally, as a post‐GWA investigation technique, BSLMM provided two new candidate QTLs for functional analyses, expanding potential breeding targets to improve cultivated *H. annuus*.

## AUTHOR CONTRIBUTIONS

J.A.D. and C.M.M. designed the study. J.A.D. assembled the phenotypic and genetic data, implemented the analyses, and interpreted the results. J.A.D. wrote the manuscript with input from C.M.M. Both authors approved the final version of the manuscript.

## Supporting information


**Appendix S1**. Gene lists for the linkage groups associated with carotenoid content in cultivated *Helianthus annuus*.

**Appendix S2**. Data analysis workflow and hyperparameter sampling results for all Bayesian sparse linear mixed and multilocus models.

## Data Availability

All supporting data are available in the Supporting Information.
