# Supplementary material for: Candidate pathway association and genome‐wide association approaches reveal alternative genetic architectures of carotenoid content in cultivated sunflower (Helianthus annuus)
Source: Appl Plant Sci. 2023 Dec 2;11(6):e11558. doi: 10.1002/aps3.11558 (PMC10719882; doi:10.1002/aps3.11558)
Supplement: Supplementary file 2 — Appendix S2. Data analysis workflow and hyperparameter sampling results for all Bayesian sparse linear mixed and multilocus models. [file APS3-11-e11558-s002.docx]

**Appendix S2.** Data analysis workflow and hyperparameter sampling results for all Bayesian sparse linear mixed models.

Abbreviations: BSLMM = Bayesian sparse linear mixed model; h = an approximation to PVE, the percent of phenotypic variance explained by genotypes; LMM = linear mixed model; N.Gamma = the number of loci with major effects, an approximation to Pi; Pi = proportion of loci with non-zero effects; PGE = percent of the percent of phenotypic variance explained by genotype; PVE = percent of phenotypic variance explained by genotype; Rho = an approximation to PGE of the percent of the percent of phenotypic variance explained by genotypes (PVE) explained by major effect loci; SNP = single-nucleotide polymorphism.

The following Supporting Information is included in this file:

Appendix S2.1. Data analysis workflow.

Appendix S2.2 (1–3). Hyperparameter sampling results for the genome-wide association study of the carotenoid content in cultivated *Helianthus annuus* using BSLMMs with genome-wide SNPS.

Appendix S2.3 (1–3). Hyperparameter sampling results for the BSLMM analysis of a genome-wide SNP subset based on the LMM results for carotenoid content in cultivated *Helianthus annuus*, consisting of the top 1% of *P* values.

Appendix S2.4 (1–3). Hyperparameter sampling results for the BSLMM analysis of a genome-wide SNP subset based on the LMM results for carotenoid content in cultivated *Helianthus annuus*, consisting of the top 1% of effect sizes.

Appendix S2.5 (1–3). Hyperparameter sampling results for the BSLMM analysis of a genome-wide SNP subset based on the LMM results for carotenoid content in cultivated *Helianthus annuus*, consisting of the top 1% of *P* values and effect sizes.

Appendix S2.6 (1–3). Hyperparameter sampling results for the BSLMM analysis of the SNPs in the carotenoid pathway, revealing their association with carotenoid content in cultivated *Helianthus annuus* (candidate pathway association).

Appendix S2.7 (1–3). Manhattan plots of multi-locus models for the analysis of a genome-wide SNP subset based on LMM results for carotenoid content in cultivated *Helianthus annuus*, consisting of the top 1% of *P* values and effect sizes.

**Appendix S2.1.** Data analysis workflow.


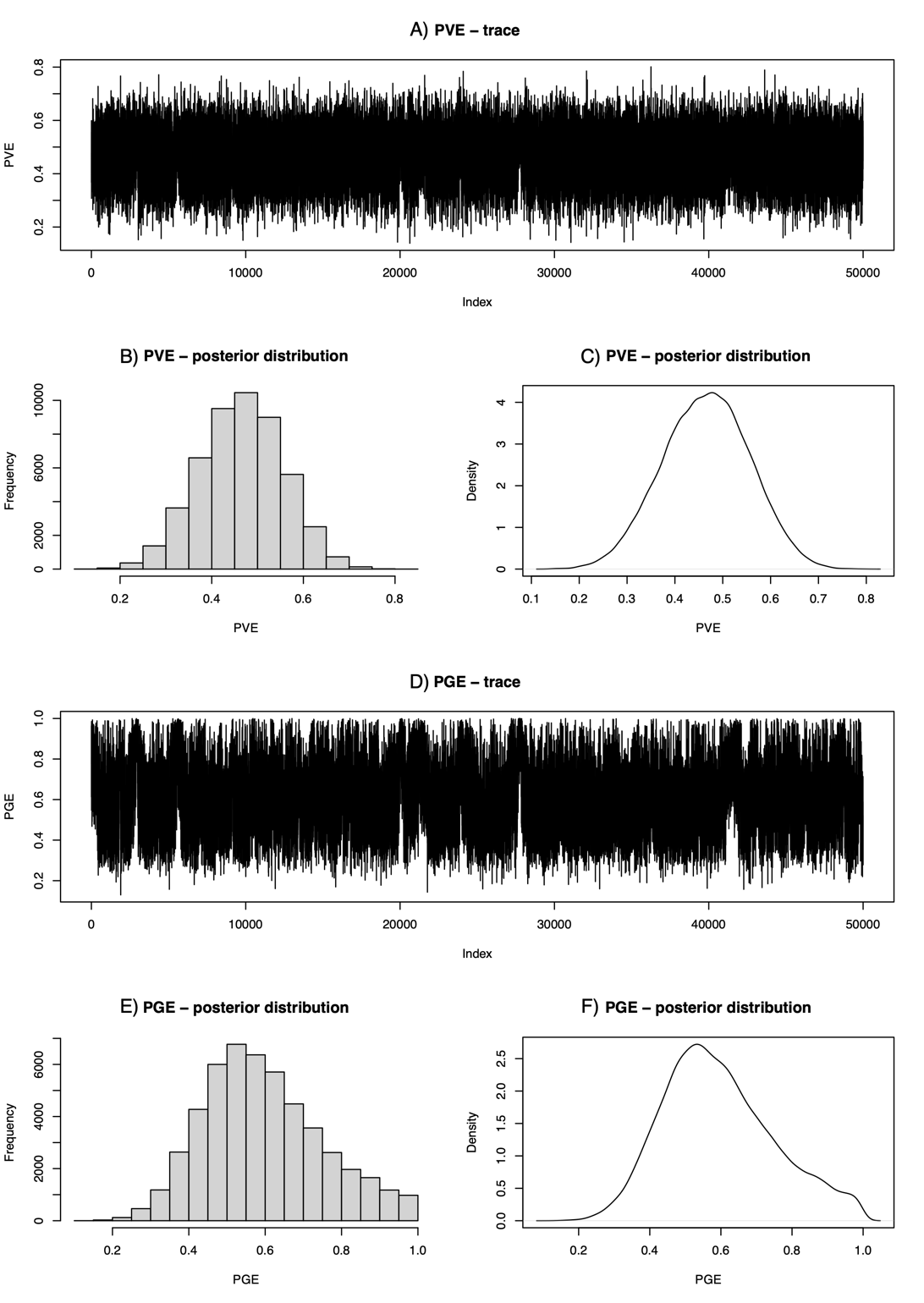


**Appendix S2.2.1.** Hyperparameter sampling results for the genome-wide association study of the carotenoid content in cultivated *Helianthus annuus* using BSLMMs with genome-wide SNPs. (A) Trace of PVE posterior sampling. (B) Histogram of the PVE posterior distribution. (C) Density plot of the PVE posterior distribution. (D) Trace of the PGE posterior sampling. (E) Histogram of the PGE posterior distribution. (F) Density plot of the PGE posterior distribution.


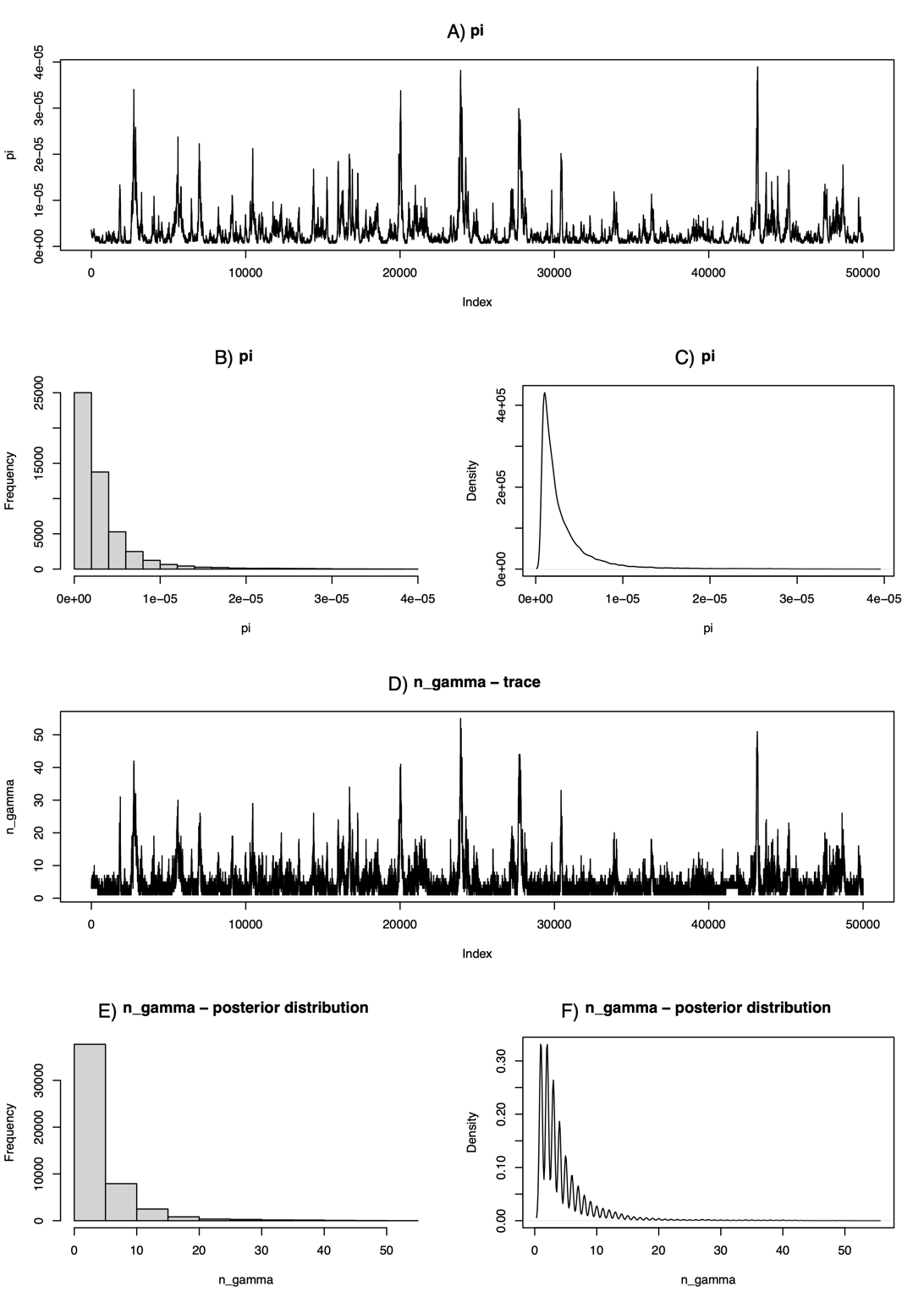


**Appendix S2.2.2.** Hyperparameter sampling results for the genome-wide association study of the carotenoid content in cultivated *Helianthus annuus* using BSLMMs with genome-wide SNPs. (A) Trace of Pi posterior sampling. (B) Histogram of the Pi posterior distribution. (C) Density plot of the Pi posterior distribution. (D) Trace of N.Gamma posterior sampling. (E) Histogram of the N.Gamma posterior distribution. (F) Density plot of the N.Gamma posterior distribution.


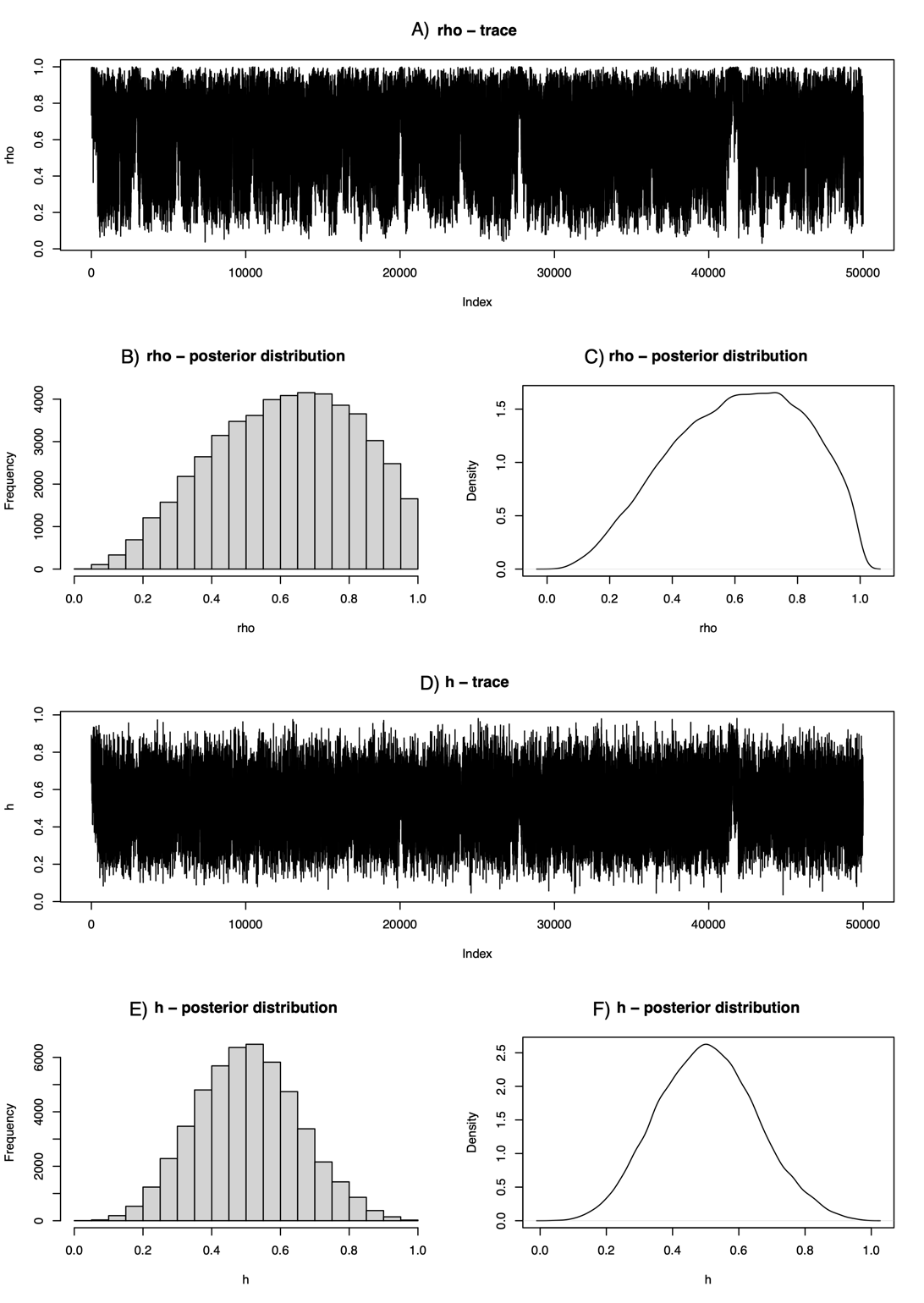


**Appendix S2.2.3.** Hyperparameter sampling results for the genome-wide association study of the carotenoid content in cultivated *Helianthus annuus* using BSLMMs with genome-wide SNPs. (A) Trace of Rho posterior sampling. (B) Histogram of the Rho posterior distribution. (C) Density plot of the Rho posterior distribution. (D) Trace of the posterior sampling of h (an approximation of PVE). (E) Histogram of the h posterior distribution. (F) Density plot of the h posterior distribution.


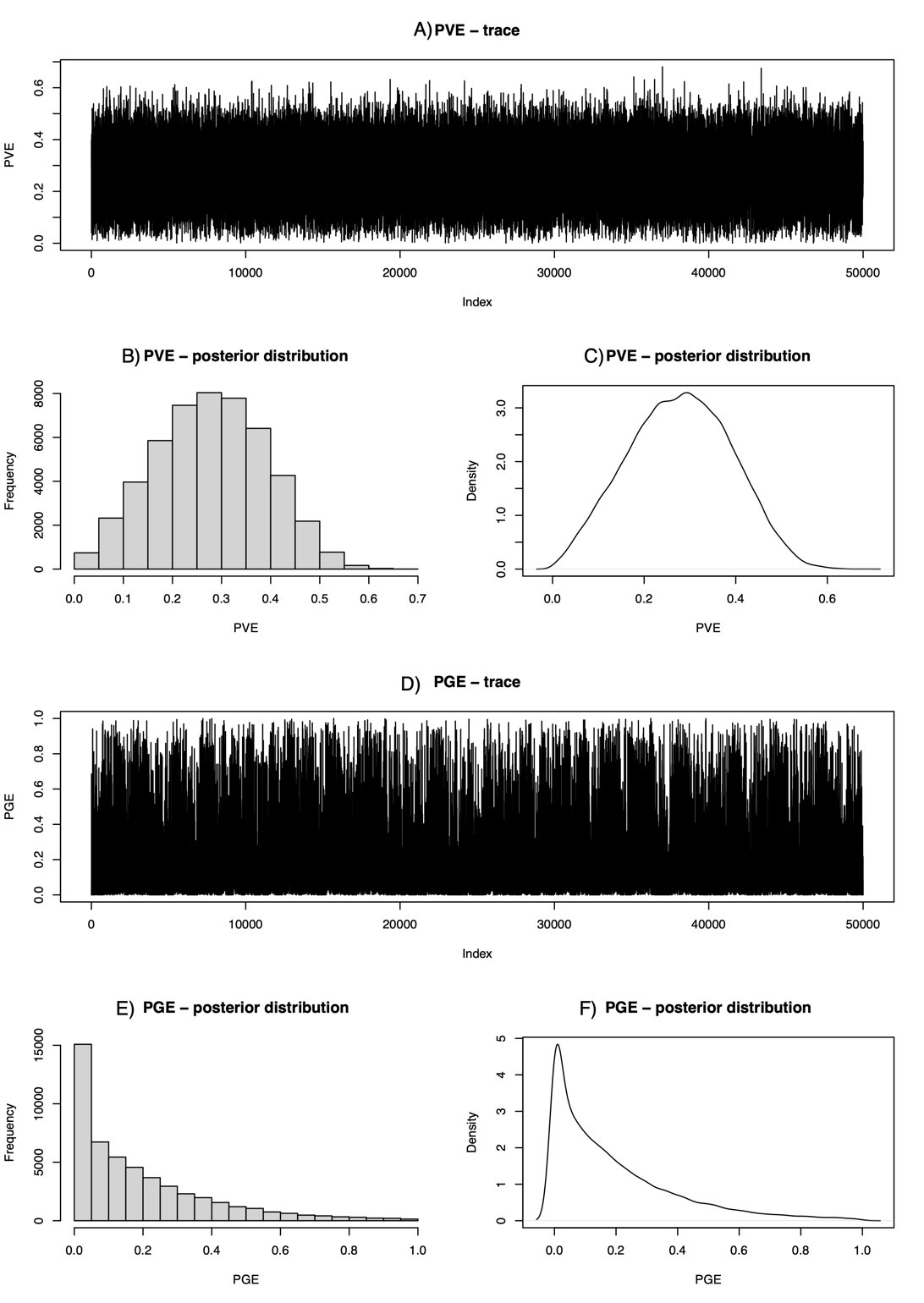


**Appendix S2.3.1.** Hyperparameter sampling results for the BSLMM analysis of a genome-wide SNP subset based on the LMM results for carotenoid content in cultivated *Helianthus annuus*, consisting of the top 1% of *P* values. (A) Trace of PVE posterior sampling (B) Histogram of the PVE posterior distribution. (C) Density plot of the PVE posterior distribution. (D) Trace of PGE posterior sampling. (E) Histogram of the PGE posterior distribution. (F) Density plot of the PGE posterior distribution.

**Appendix S2.3.2.** Hyperparameter sampling results for the BSLMM analysis of a genome-wide SNP subset based on the LMM results for carotenoid content in cultivated *Helianthus annuus*, consisting of the top 1% of *P* values. (A) Trace of Pi posterior sampling. (B) Histogram of the Pi posterior distribution. (C) Density plot of the Pi posterior distribution. (D) Trace of N.Gamma posterior sampling. (E) Histogram of the N.Gamma posterior distribution. (F) Density plot of the N.Gamma posterior distribution.

**Appendix S2.3.3.** Hyperparameter sampling results for the BSLMM analysis of a genome-wide SNP subset based on the LMM results for carotenoid content in cultivated *Helianthus annuus*, consisting of the top 1% of *P* values. (A) Trace of Rho posterior sampling. (B) Histogram of the Rho posterior distribution. (C) Density plot of the Rho posterior distribution. (D) Trace of h posterior sampling. (E) Histogram of the h posterior distribution. (F) Density plot of the h posterior distribution.

**Appendix S2.4.1.** Hyperparameter sampling results for the BSLMM analysis of a genome-wide SNP subset based on the LMM results for carotenoid content in cultivated *Helianthus annuus*, consisting of the top 1% of effect sizes. (A) Trace of the PVE posterior sampling. (B) Histogram of the PVE posterior distribution. (C) Density plot of the PVE posterior distribution. (D) Trace of the PGE posterior sampling. (E) Histogram of the PGE posterior distribution. (F) Density plot of the PGE posterior distribution.

**Appendix S2.4.2.** Hyperparameter sampling results for the BSLMM analysis of a genome-wide SNP subset based on the LMM results for carotenoid content in cultivated *Helianthus annuus*, consisting of the top 1% of effect sizes. (A) Trace of Pi posterior sampling. (B) Histogram of the Pi posterior distribution. (C) Density plot of the Pi posterior distribution. (D) Trace of N.Gamma posterior sampling. (E) Histogram of the N.Gamma posterior distribution. (F) Density plot of the N.Gamma posterior distribution.

**Appendix S2.4.3.** Hyperparameter sampling results for the BSLMM analysis of a genome-wide SNP subset based on the LMM results for carotenoid content in cultivated *Helianthus annuus*, consisting of the top 1% of effect sizes. (A) Trace of Rho posterior sampling. (B) Histogram of the Rho posterior distribution. (C) Density plot of the Rho posterior distribution. (D) Trace of h posterior sampling. (E) Histogram of the h posterior distribution. (F) Density plot of the h posterior distribution.

**Appendix S2.5.1.** Hyperparameter sampling results for the BSLMM analysis of a genome-wide SNP subset based on the LMM results for carotenoid content in cultivated *Helianthus annuus*, consisting of the top 1% of *P* values and effect sizes. (A) Trace of PVE posterior sampling. (B) Histogram of the PVE posterior distribution. (C) Density plot of the PVE posterior distribution. (D) Trace of PGE posterior sampling. (E) Histogram of the PGE posterior distribution. (F) Density plot of the PGE posterior distribution.

**Appendix S2.5.2.** Hyperparameter sampling results for the BSLMM analysis of a genome-wide SNP subset based on the LMM results for carotenoid content in cultivated *Helianthus annuus*, consisting of the top 1% of *P* values and effect sizes. (A) Trace of Pi posterior sampling. (B) Histogram of the Pi posterior distribution. (C) Density plot of the Pi posterior distribution. (D) Trace of N.Gamma posterior sampling. (E) Histogram of the N.Gamma posterior distribution. (F) Density plot of the N.Gamma posterior distribution.

**Appendix S2.5.3.** Hyperparameter sampling results for the BSLMM analysis of a genome-wide SNP subset based on the LMM results for carotenoid content in cultivated *Helianthus annuus*, consisting of the top 1% of *P* values and effect sizes. (A) Trace of Rho posterior sampling. (B) Histogram of the Rho posterior distribution. (C) Density plot of the Rho posterior distribution. (D) Trace of h posterior sampling. (E) Histogram of the h posterior distribution. (F) Density plot of the h posterior distribution.

**Appendix S2.6.1.** Hyperparameter sampling results for the BSLMM analysis of the SNPs in the carotenoid pathway, revealing their association with carotenoid content in cultivated *Helianthus annuus* (candidate pathway association). (A) Trace of PVE posterior sampling. (B) Histogram of the PVE posterior distribution. (C) Density plot of the PVE posterior distribution. (D) Trace of PGE posterior sampling. (E) Histogram of the PGE posterior distribution. (F) Density plot of the PGE posterior distribution.

**Appendix S2.6.2.** Hyperparameter sampling results for the BSLMM analysis of the SNPs in the carotenoid pathway, revealing their association with carotenoid content in cultivated *Helianthus annuus* (candidate pathway association). (A) Trace of Pi posterior sampling. (B) Histogram of the Pi posterior distribution. (C) Density plot of the Pi posterior distribution. (D) Trace of N.Gamma posterior sampling. (E) Histogram of the N.Gamma posterior distribution. (F) Density plot of the N.Gamma posterior distribution.

**Appendix S2.6.3.** Hyperparameter sampling results for the BSLMM analysis of the SNPs in the carotenoid pathway, revealing their association with carotenoid content in cultivated *Helianthus annuus* (candidate pathway association). (A) Trace of Rho posterior sampling. (B) Histogram of the Rho posterior distribution. (C) Density plot of the Rho posterior distribution. (D) Trace of h posterior sampling. (E) Histogram of the h posterior distribution. (F) Density plot of the h posterior distribution.

A)

B)

**Appendix S2.7.1.** Manhattan plots of multi-locus models for the analysis of a genome-wide SNP subset based on LMM results for carotenoid content in cultivated *Helianthus annuus*, consisting of the top 1% of *P* values. Results from (A) Farm-CPU and (B) BLINK.

A)

B)

**Appendix S2.7.2.** Manhattan plots of multi-locus models for the analysis of a genome-wide SNP subset based on LMM results for carotenoid content in cultivated *Helianthus annuus*, consisting of the top 1% of effect sizes. Results from (A) Farm-CPU and (B) BLINK.

A)

B)

**Appendix S2.7.3.** Manhattan plots of multi-locus models for the analysis of a genome-wide SNP subset based on LMM results for carotenoid content in cultivated *Helianthus annuus*, consisting of the top 1% of *P* values and effect sizes. Results from (A) Farm-CPU and (B) BLINK.
